# Structure of Nanobody Nb23

**DOI:** 10.3390/molecules26123567

**Published:** 2021-06-11

**Authors:** Mathias Percipalle, Yamanappa Hunashal, Jan Steyaert, Federico Fogolari, Gennaro Esposito

**Affiliations:** 1 Science Division, New York University Abu Dhabi, Abu Dhabi 129188, United Arab Emirates; mp5604@nyu.edu (M.P.); yh45@nyu.edu (Y.H.); 2 Department of Chemistry and Magnetic Resonance Center, University of Florence, 50019 Florence, Italy; 3 Structural Biology Brussels, Vrije Universiteit Brussel, B-1050 Brussels, Belgium; jan.steyaert@vub.be; 4 VIB-VUB Center for Structural Biology, Vlaams Instituut voor Biotechnologie, B-1050 Brussels, Belgium; 5 Dipartimento di Scienze Matematiche, Informatiche, e Fisiche, Udine University, 33100 Udine, Italy; federico.fogolari@uniud.it; 6 Istituto Nazionale Biostrutture e Biosistemi, 00136 Roma, Italy

**Keywords:** nanobody, protein structure, immunoglobulin domain, NMR

## Abstract

Background: Nanobodies, or VHHs, are derived from heavy chain-only antibodies (hcAbs) found in camelids. They overcome some of the inherent limitations of monoclonal antibodies (mAbs) and derivatives thereof, due to their smaller molecular size and higher stability, and thus present an alternative to mAbs for therapeutic use. Two nanobodies, Nb23 and Nb24, have been shown to similarly inhibit the self-aggregation of very amyloidogenic variants of β2-microglobulin. Here, the structure of Nb23 was modeled with the Chemical-Shift (CS)-Rosetta server using chemical shift assignments from nuclear magnetic resonance (NMR) spectroscopy experiments, and used as prior knowledge in PONDEROSA restrained modeling based on experimentally assessed internuclear distances. Further validation was comparatively obtained with the results of molecular dynamics trajectories calculated from the resulting best energy-minimized Nb23 conformers. Methods: 2D and 3D NMR spectroscopy experiments were carried out to determine the assignment of the backbone and side chain hydrogen, nitrogen and carbon resonances to extract chemical shifts and interproton separations for restrained modeling. Results: The solution structure of isolated Nb23 nanobody was determined. Conclusions: The structural analysis indicated that isolated Nb23 has a dynamic CDR3 loop distributed over different orientations with respect to Nb24, which could determine differences in target antigen affinity or complex lability.

## 1. Introduction

Single-domain antibodies, or nanobodies, are derived from heavy-chain only antibodies (HcAbs) found in camelids [1]. Essentially, they can be used for the same therapeutic purposes as monoclonal antibodies (mAbs) and single-chain variable fragments (scFvs) but with some advantages brought about by their inherent properties. For one, the small molecular size of nanobodies (~15 kDa) facilitates penetrance to target sites, as nanobodies are half as large as scFvs and five times smaller than human conventional antibodies [2]. This, in combination with more extended loops of the complementarity determining regions 1 and 3 (CDR1 and CDR3), enables binding to a wider range of epitopes with different shapes at sub-nanomolar affinity, potentially increasing the application of nanobodies as drugs. The lack of a light chain in HcAbs also allows nanobodies to exist as a single domain with less susceptibility to aggregation through hydrophobic interactions, as is the case for scFvs [3,4,5]. Due to their small size and high similarity to the human immunoglobulin variable domain, they provoke little to no immune response [5] which often makes humanization unnecessary.

Amyloidogenic proteins have previously been targeted with nanobodies to inhibit the course of amyloidogenesis [4]. Nanobodies have been shown to inhibit the formation of amyloid β (Aβ) fibrils formed in Alzheimer’s disease patients, and also to recognize non-conventional epitopes on Aβ fibrils for diagnostic use [6], although the clinical trials to validate antibody drugs have been unsuccessful so far.

Non-neurodegenerative amyloidoses may prove more amenable for nanobody treatment. A paradigmatic amyloidogenic protein, β2-microglobulin (β2m), which is a component of class I major histocompatibility complex (MHC-1), accumulates as amyloid deposits in the joints of patients undergoing long-term haemodialysis [7]. The deposits contain some 30% of ΔN6β2m, the proteolytic variant of β2m devoid of the N-terminal hexapeptide, that forms fibrils also by mild stirring at neutral pH [8]. This amyloidogenic propensity, much stronger than the parent protein, was also observed with D76Nβ2m, a naturally occurring variant of β2m that causes progressive bowel dysfunction and systemic amyloidosis, i.e., deposits in several vital organs [9].

Several nanobodies were raised against wild-type (WT) β2m and ΔN6β2m by immunization of both a camel and a llama. Nb24, a camel-derived nanobody raised against WT β2m has been shown to inhibit the self-aggregation of the very amyloidogenic ΔN6β2m and D76Nβ2m variants in vitro and, indirectly, also in vivo, and the binding thermodynamics and kinetics along with the epitope mapping of the D76Nβ2m-Nb24 complex were characterized [10,11]. In this case, D76Nβ2m self-aggregation was inhibited despite the fact that Nb24 was raised against the WT β2m. The crystal structure of Nb24 complexes with ΔN6β2m (PDB ID 2X89) and P32Gβ2m (PDB ID 4KDT) are known [11,12] whereas no structure is available for the isolated nanobody. Nb23, which is instead llama-derived and raised against ΔN6β2m, inhibits self-aggregation of its raising antigen, but fails to inhibit D76Nβ2m self-aggregation, despite it being raised against a very amyloidogenic variant of β2m. In order to characterize the interaction of Nb23 with a target other than the original antigen, structural information is crucial. In this study, the solution structure of Nb23 has been determined using nuclear magnetic resonance (NMR) spectroscopy, as a first step of a general project aimed at rationalizing the determinants of nanobody performance with β2m variants. In particular, structure knowledge enables systematic analysis of the conformational, thermodynamic, and kinetic properties of the binding to the β2m variants in order to improve the affinity between nanobody and antigen or attenuate their complex lability through rational design.

## 2. Results

### 2.1. Nb23 Sequence Inferences

The Nb23 construct characterized here consists of 136 amino acids, including an initial methionine residue introduced as a start codon and therefore referred to as Met0, and a (His)_6_ tag at the C-terminus of the protein for expression in *E. coli* and purification, amounting to a molecular weight of 15.1 kDa. There are two cysteines at position 22 and 96 which form the disulfide bond between the two β-sheets of the expected immunoglobulin domain. Nb23 and Nb24 are of equal lengths with 71% identity, and 75% positive identity. This level of homology indicates structural and functional similarity [13]. The fact that the main variation in sequences between Nb23 and Nb24 coincides with the CDRs (located between residues 26–32, 52–57, and 100–116), together with a general consensus on the typical structural similarity of the framework regions of immunoglobulin variable domains, suggests that the frameworks of both nanobodies are similar.

### 2.2. NMR Spectroscopy Results and Chemical Shift Assignment Completeness

**Figure 1:**
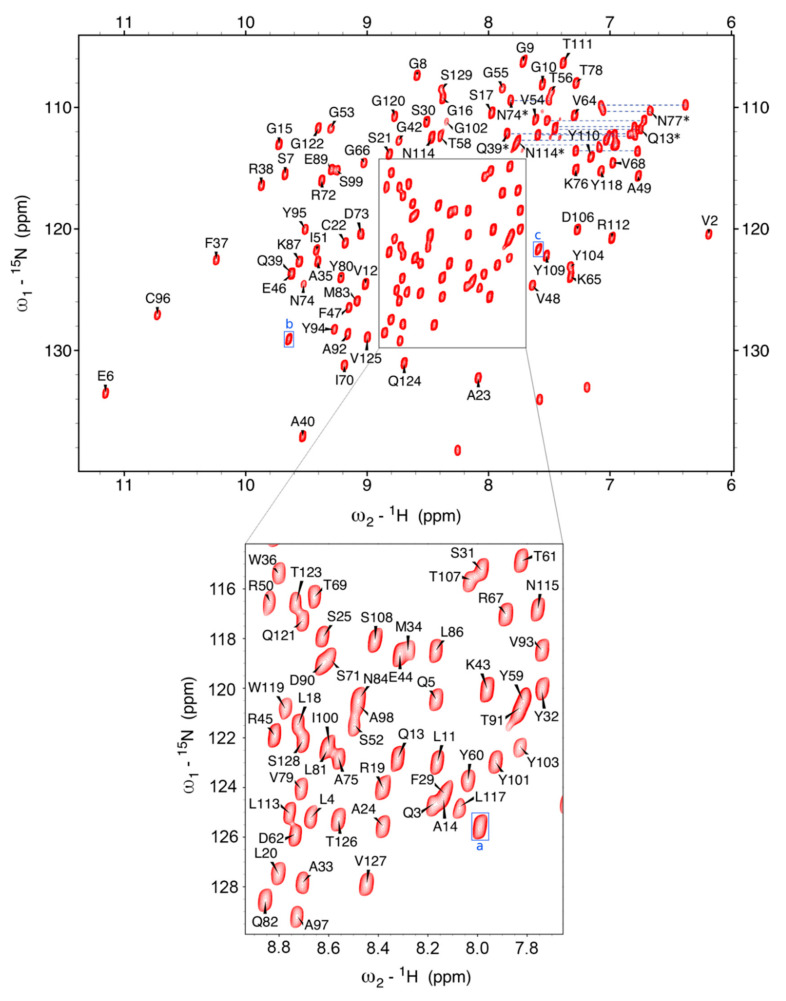
The ^15^N−^1^H HSQC of Nb23 from a freshly prepared sample (247 µM in 19.5 mM bis-Tris and 21 mM NaCl). The good signal−to−noise of the spectrum allowed the application of a squared sine−bell shifted by π/6 to achieve complete resolution. Excluding Met0 and the C−terminal (His)_6_ tag used for expression, five N−H connectivities could not be assigned (Gln1, Arg27, Thr28, Ser63, and Ser105). Only the three blue-boxed connectivities, labeled a, b, and c, out of those that were observed, could not be attributed through scalar correlation. A tentative assignment is proposed based on heteronuclear NOE (see main text). The central area highlighted with a box has been enlarged for better visualization (lower panel) to limit the assignment annotation crowding given the high density of peaks. The Asn and Gln sidechain carboxyamide pairs could be connected from the slow exchange cross−peak of 2D ^1^H−^1^H NOESY, which also enabled the identification in a few cases from intra−residue NOE. The pairs are connected with blue dashed lines and the assigned ones are marked with an asterisk. The dispersion of peaks indicates a well−structured protein. The remaining peaks without labels belong to sidechain NHs, i.e., Arg, His, and Trp.

**Figure 2:**
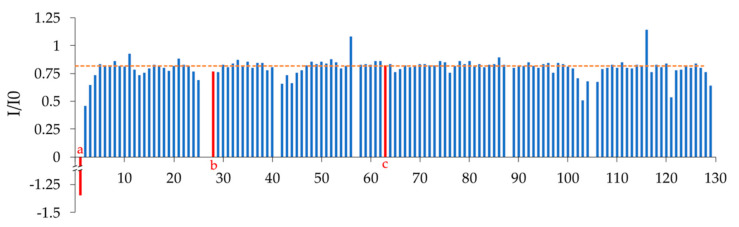
^15^N{^1^H} NOE values, with I/I0 ratios representing the individual amide signal intensity with and without hydrogen saturation. The horizontal dotted line marks the average ratio value. Ratios below the average line indicate regions of mobility in the protein. The main regions of flexibility correspond to the supposed CDR1 (positions 26−31), a supposed loop between positions 42 and 45, and the supposed initial part of the CDR3 (positions 102−106). Residues with no bar correspond to either prolines (Pr041 and Pro88) or residues which were missing NH assignment. Based on the NOE values obtained for peaks (a), (b), and (c), that did not show scalar correlation in 3D spectra (Figure 1), a tentative assignment is proposed, respectively Gln1, Thr28, and Ser63, as indicated by the positions of the red bars.

The ^15^N-^1^H HSQC spectrum of Nb23 is shown in Figure 1. The resonance spreading already appears quite satisfactory, and TROSY pulse schemes further enabled the resolution of certain overlapping peaks in the regular ^15^N-^1^H HSQC. Apart from the two prolines which lack amide protons and excluding Met0 and the (His)_6_ tag, amide connectivity assignments are missing for Gln1, Arg27, Thr28, Ser63, and Ser105, which include residues of the expectedly mobile CDR1 (Arg27 and Thr28) and CDR3 (Ser105) loops. The occurrence of conformational mobility at intermediate rate on the chemical shift scale leading to signal broadening seems confirmed by the fact that neighboring residues in CDR1 and CDR3 (Gly26 and Gly102) exhibit below-average intensities and by the ^15^N{^1^H} NOE data, where residues in conformationally rigid regions show a close-to-average ratio of peak intensity with and without hydrogen saturation (Figure 2). It is thus plausible that an unfavorable conformational exchange rate in the CDR regions could affect the detectability of some signal in ^15^N-^1^H HSQC and TROSY spectra. On the other hand, the unassigned peaks other than sidechain resonances that were observed in the ^15^N-^1^H HSQC or TROSY maps—namely three cross-peaks highlighted by blue boxes and letter labels in Figure 1—were addressed, but no conclusion could be achieved through the correlation patterns of the 3D triple resonance experiments acquired for backbone assignment, suggesting again that some slow conformational exchange occurring over the ms-to-μs time scale accelerates relaxation, thereby hindering the propagation of the coherence transfer pathway. The extent of population transfer from ^15^N{^1^H} NOE data (Figure 2) enables, however, a tentative assignment. The negative heteronuclear NOE of boxed peak (a) is very likely to arise from Gln1. The close-to-average NOE value of boxed peak (c) could be consistent with the mobility expected at Ser63. Finally, the NOE value observed for boxed peak (b) suggests a possible attribution to Thr28, given the similar NOE value measured at Phe29. This dipolar-coupling-based assignment leaves only Arg27 (CDR1) and Ser105 (CDR3) without observable ^15^N-^1^H connectivity signal that, in turn, corresponds to the signature of a conformational exchange process at the start of CDR1 and CDR3.

Typical TROSY-based 3D triple resonance spectra [14,15] (see Section 4) were used to assign the backbone and sidechain atoms. The sidechain assignment was arduous especially for residues with very long sidechains, due to the relaxation attenuation ensuing from many magnetization transfers combined with the relatively low sample concentrations, leading to noisy data with reduced intensity. The low sample concentrations were in turn due to poor protein solubility, at least for the particular sample conditions used here, and concentrations were further reduced by the subsequent protein precipitation occurring during the data acquisition.

The aromatic sidechain hydrogen atoms of Tyr, Phe, and Trp residues were assigned using the 2D experiments correlating the Hδ and Hε to the Cβ (2D CBHD and CBHE [16]) with samples in 100% D_2_O. The corresponding aromatic carbons were identified in the ^13^C-^1^H HSQC. Due to extensive overlap of the aromatic carbon atoms in the spectra, only 32% of them could be assigned unambiguously.

The total percentages of chemical shifts assigned are reported in Table 1. Excluding Met0, the (His)_6_ tag and two Pro residues, the backbone assignments (Cα, C’, HN, N and Hα) were 95% complete, the sidechain residue assignments (including Cβ and Hβ) were 67% complete, and the aromatic residue assignments were 50% complete. Overall, the chemical shift assignment was achieved to an extent of 77%. The majority of the unassigned chemical shifts for both backbone and sidechain belong to residues of the CDR1 and CDR3 regions, which are expectedly less rigid than the remaining structure, thereby leading to inherently poor frequency spreading and/or broad line widths when unfavorable mobility rates are also involved. The completeness limits of the aromatic residue assignment could instead be totally ascribed to extensive resonance degeneracy from high mobility, for which characterization was mostly ambiguous and hence peaks unassignable, especially for carbons.

### 2.3. Secondary Structure Content Assessment

**Figure 3:**
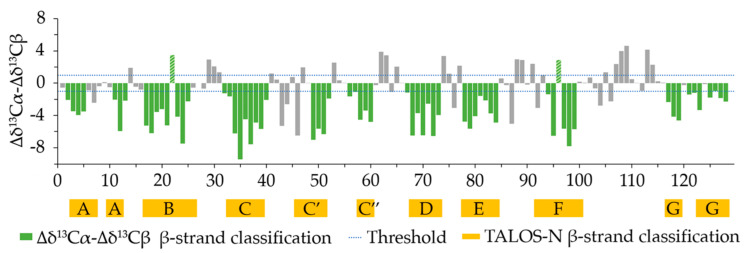
The chemical shift indexing analysis (CSI), computed by taking the difference between the experimentally determined Cα chemical shifts and the Cα random coil chemical shift (∆δ^13^Cα) minus the difference between the experimentally determined Cβ chemical shift and the Cβ random coil chemical shift (∆δ^13^Cβ). Three negative ∆δ^13^Cα − ∆δ^13^Cβ values in a row indicate the presence of β-strand. A cumulative threshold error based on the individual ∆δ deviations of ±0.7 ppm, i.e., $\sqrt{\left(0.7^{2}+0.7^{2}\right)}≅1\;$ ppm, was used as a threshold to include only significantly varying consecutive negative values. Residues predicted to be in β−strands are highlighted in green in the graph. The chemical shift differences of Cys22 and Cys96 (highlighted by green hatched bars) deviate because of upfield shifts induced by aromatic sidechains. As a consequence, especially for the Cβ chemical shifts, typical values of the reduced cysteines were observed despite the presence of the disulfide bridge with the associated β structure content. Control CD spectra of oxidized and reduced Nb23 are reported in Supplementary Materials to illustrate the issue, showing that Cys22 and Cys96 form a disulfide bridge. Yellow blocks indicate the position of residues that were estimated to be in β−strands by TALOS-N.

An assessment of secondary structure content was made by looking at the difference of the deviations from random conformation chemical shifts of the assigned Cα and Cβ resonances (∆δ^13^Cα − ∆δ^13^Cβ) [17]. To identify secondary structure elements using the individual carbon resonances, the chemical shifts are compared to the random coil chemical shift of the corresponding residue. A difference larger than ±0.7 ppm from the random coil chemical shift for several consecutive residues indicates the presence of secondary structure elements. Four consecutive downfield shifted Cα resonances beyond the 0.7 ppm threshold with respect to the random coil shift indicate α-helical structure, while three consecutive upfield shifted resonances in a row indicate β-strand presence. The opposite is true for Cβ resonances (downfield shift indicates β-strand, upfield shift indicates α-helix) [18]. The difference between the ∆δ^13^Cα and Δδ^13^Cβ eliminates any possible chemical shift reference error on the individual deviations, with a positive ∆δ^13^Cα − Δδ^13^Cβ difference indicating α-helix and a negative difference indicating β-strand. Here, a cumulative approach to identify secondary structure elements from the ∆δ^13^Cα − Δδ^13^Cβ difference was employed by using an error threshold derived from the individual ± 0.7 ppm deviations of ∆δ^13^Cα and Δδ^13^Cβ, i.e., $\sqrt{\left(0.7^{2}+0.7^{2}\right)}≅1\;$ ppm. The results are illustrated in Figure 3, with the expected secondary structure elements highlighted in the figure. Overall, nine β-segments could be identified, a number consistent with the typical β-strand content of a canonical immunoglobulin variable domain, with a percentage of residues involved in β-strands of 49.6%. In comparison, Nb24 has a β-strand content of 50.4% when bound to antigen [11]. One possible α-helical tract was identified in the supposed CDR3 loop between residues 107 and 109.

For an alternative assessment of secondary structure content, TALOS-N [19] was also used to infer φ and ψ torsion angles of Nb23 sequence from its backbone and Cβ chemical shift assignments. Torsion angles are in turn characteristic for certain types of secondary structures. The secondary structure content obtained by TALOS-N assessment is also illustrated in Figure 3. Here β-strand content was also 50.4% (as for Nb24), marking a difference with the chemical shift indexing analysis.

Circular dichroism (CD) data collected for Nb23 and uploaded to the Beta Structure Selection (BeStSel) server, a CD data analysis server especially useful for identification of β structures [20], show that Nb23 is mainly composed of antiparallel β-strands with different twists. No α-helical segments were identified. The overall β-strand content of the structure was 55.2%, which is slightly exceeding the content from the chemical shift indexing and TALOS-N estimations. This is not surprising as BeStSel assessment also includes relaxed β-strands. The results from the BeStSel analysis can be found in the Supplementary Materials.

### 2.4. Constraints and Nb23 Structure Calculation

Given the lack of assignment for a number of Nb23 sidechain resonances, an alternative strategy was employed to collect necessary constraints for restrained modeling. The CS-Rosetta server was used to provide a model for Nb23 in order to facilitate the search for experimental constraints. CS-Rosetta uses chemical-shift-constrained homology modeling to outline a 3D protein structure, based on the prediction of backbone and side-chain dihedral angles from the amino-acid sequence and the analogy of the experimental chemical shifts with those of a characterized model ensemble derived from PDB and BMRB [21]. The CS-Rosetta run generated 40,000 models of Nb23. The Cα-Root Mean Square Deviation (Cα-RMSD) was calculated for all of the models with respect to the lowest energy structure, yielding an averaged Cα-RMSD of 1.53 ± 0.99 Å for the ten best structures, calculated over the fragments 1–102, 117–122. Residues 103–116, coinciding with the tentative location of CDR3 loop, were considered as a flexible region. The CS-Rosetta run was deemed as successful as it achieved a Cα-RMSD below 2 Å for non-flexible regions for the ten lowest energy structures and the run converged towards a single structure.

The average β-structure content of the CS-Rosetta models was 49.2%, comparable to the β-structure content of TALOS-N and CD. The β-strand positions also coincided well with the TALOS-N β-strand positions except between residues 57 and 60, where β-secondary structure was consistently absent in the models.

Given the good agreement between the TALOS-N estimates, CD spectroscopy results, and the CS-Rosetta models regarding the β-secondary structure content, as well as the satisfactory Cα-RMSD for the ten best structures, the CS-Rosetta models were deemed as representative of Nb23 for the residues 1–102 and 117–122, and used as prior knowledge for NOE-constraint identification. The conformation of the CDR3 (residues ~101–116) was however not defined for the CS-Rosetta models and was not used for the same purpose.

A 3D ^15^N-^1^H NOESY HSQC spectrum, and aliphatic and aromatic 3D ^13^C-^1^H NOESY HSQC spectra, were acquired in order to extract NOE constraints for structure determination. Complementary 2D ^1^H-^1^H NOESY spectra were also acquired using unlabeled protein samples. Besides the attribution difficulties deriving from the missing sidechain assignments, the NOE identification was also hampered by resonance overlap and critical signal-to-noise ratio due to progressive decrease of protein concentration. The total number of NOE constraints extracted from the spectra using automated and manual assignments, handled by means of the software PONDEROSA [22,23], with prior knowledge from CS-Rosetta models was limited (619), first because of the lack of extensive assignment for the aliphatic and aromatic sidechains, and second because of selection of only unequivocal correlations. This apparently “minimalist” approach was adopted because the structural restraining was already based on the experimentally constrained models of CS-Rosetta, that included 734 chemical shift values constraining 353 dihedral angles. Nonetheless, very characteristic NOE patterns for β-secondary structure types [24] concerning backbone atoms were identified for most residues expected to be found in β-strands as per the chemical shift indexing analysis. Hydrogen bonded amides were also identified by recording a ^15^N-^1^H HSQC spectrum one week after transferring the protein to D_2_O. This allowed for identification of slowly exchanging amide protons which are involved in secondary structure formation or are otherwise hydrogen bonded [25]. In that spectrum, the backbone NHs of 18 residues were characterized as slowly exchanging, all of which were expected to occur in secondary structure elements as per the chemical shift indexing analysis. The corresponding H-bonds were thus added as distance restraints (the relative list is reported in Supplementary Materials, Table S1). The 20 best NOE-restrained structures were validated with the tools of the PDB Validation Service [26,27,28] (see Supplementary Materials) and subjected to energy minimization as described in the Materials and Methods section. The ensemble of the ten lowest energy and most similar structures was retained. The relative validation report can be found in the Supplementary Materials.

A summary of the structural features and violations of the CS-Rosetta ensemble, the 20 NOE-restrained structures, and the ten NOE-restrained energy-minimized ensemble is shown in Table 2.

### 2.5. Nb23 Structural Features

**Figure 4:**
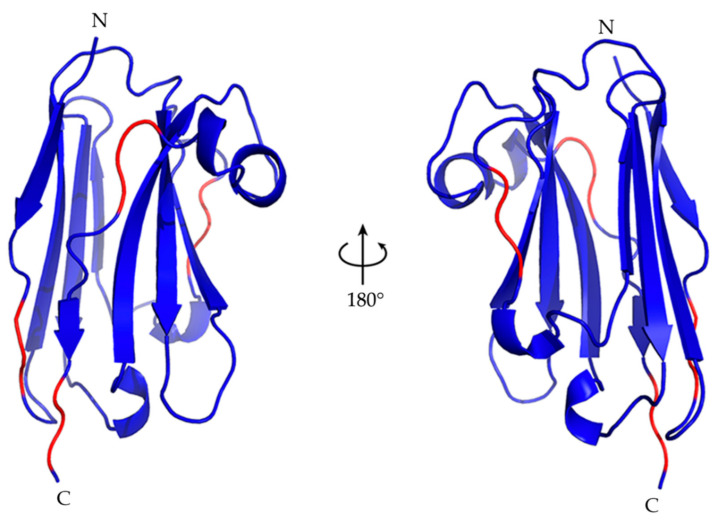
The best Nb23 structure from energy minimization of the NOE-restrained PONDEROSA C/S models. The structure is the lowest energy conformer of the NOE-restrained best cluster deposited in PDB (7EH3). It has the general features of a variable immunoglobulin domain, with the characteristic extended CDR3 of nanobodies which for Nb23 shields the solvent-exposed hydrophobic sidechains of Phe37, Phe47, Ile51, and Trp119. The β-strand content in the NOE-restrained best cluster is under-represented with respect to the analogous content of the CS-Rosetta structure ensemble. The red color highlights the location of the fragments extended but devoid of regular β-structure. Table 3 shows the positions of the β-strands for each structure of the NOE-restrained best cluster.

**Figure 5:**
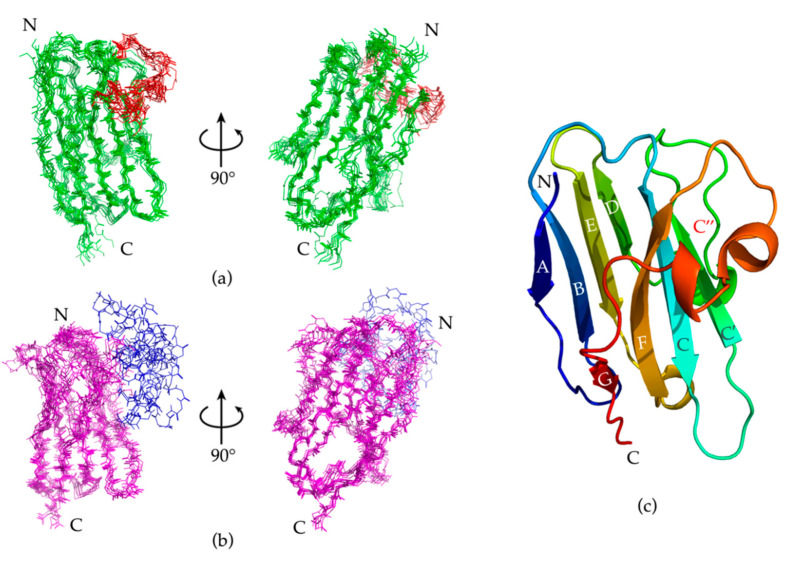
(**a**) An overlay of the Nb23 backbone of the NOE-restrained best cluster. The Cα−RMSD with respect to the best structure was 1.57 ± 0.32 Å, with substantial conformational dispersion localized in the CDR3 (highlighted in red). By excluding the CDR3 and residues 1, 2, and 129 from the alignment, the Cα−RMSD was 1.23 ± 0.30 Å. (**b**) An overlay of the Nb23 backbone of the CS−Rosetta ensemble. The Cα−RMSD with respect to the lowest energy structure was 3.42 ± 2.12. By excluding the CDR3 (highlighted in blue), the Cα-RMSD was 1.53 ± 0.99 Å, calculated over the fragments 1−102, 117−122. The conformational dispersion at the CDR3 is much more pronounced than the spread of the corresponding region in the NOE-restrained best cluster. (**c**) A visualization of the positions of the β−strands, lettered in white or grey. The only whole strand missing (C’’) is highlighted in red, and protein terminals in black.

The ten best Nb23 structures from energy minimization were deposited in the PDB (PDB ID 7EH3) and will be henceforth referred to as NOE-restrained best cluster. The first structure of the NOE-restrained best cluster is shown in Figure 4. The dispersion of the structures within this cluster was assessed by Cα-RMSD. The averaged Cα-RMSD with respect to the best structure was 1.57 ± 0.32 Å. Excluding the CDR3 (residues 101–117), which is expectedly more mobile and is the most variable part of immunoglobulin domains, and residues 1, 2, and 129, the Cα-RMSD was instead 1.23 ± 0.30 Å, highlighting the extent of the CDR3 contribution. An overlay of the backbone of the NOE-restrained best cluster is shown in Figure 5a. The corresponding β-structure content detailed in Table 3 for each element of the cluster can be compared to the experimental data from the ∆δ^13^Cα − Δδ^13^Cβ chemical shift indexing analysis and the TALOS-N assessment of secondary structure content shown in Figure 3. The superposition of the CS-Rosetta ensemble displayed in Figure 5b highlights the much larger dispersion of the CDR3 region with respect to the NOE-restrained best cluster. A visualization of the positions of the β-strands is shown in Figure 5c. The average β-structure content of the NOE-restrained best cluster is 40.9%, which is lower with respect to the CSI and TALOS-N estimations. Structure 3 (43.4% β-structure content) and Structure 8 especially (46.5% β-structure content) exhibit better and very similar overlap with the CSI, TALOS-N and CS-Rosetta models, while the remaining conformers of the ensemble have a more lacking β-structure content to the one inferred from the CSI and TALOS-N. It is possible that proper β-structure did not appear in the fragments highlighted in Figure 4 due to the relatively low number of constraints found for Nb23. Given that both the β-strand content scores from CSI, TALOS-N and CS-Rosetta modeling indicate higher values, in analogy with the evidence from CD, the β-structure content of the NOE-restrained best cluster may be underestimated. However, the absence of inter-strand NOEs, especially at the edges of the sheets, concerning primarily backbone residues, also suggests the occurrence of loose geometry in solution, as observed with isolated immunoglobulin motifs in solution [8,10].

A different assessment of this scenario may come from an evaluation of the structural data that were obtained by CS-Rosetta or NOE-restrained and energy minimization modeling, based on the recently proposed ANSURR method [29]. According to this validation approach, the accuracy of an NMR structure cannot be inferred from the spread of the final conformation ensemble, which reflects only the precision of the determination. The structural dispersion must be coupled to the correlation between the CSI and the flexibility of the molecule, as scored by software suites that exploit prior knowledge from data banks and/or neural networks. The ANSURR evaluation tested on decoys and real structures shows an interesting diversification between prevalently helical proteins and prevalently β proteins, with the former exhibiting a much higher flexibility-CSI correlation score than RMSD score, and the latter showing the opposite, i.e., a higher RMSD score than flexibility-CSI correlation. The ANSURR evaluation of the CS-ROSETTA ensemble appears to feature somehow the characteristics of the prevalently β-structured proteins, with average correlation and RMSD average scores of 24 ± 15 and 89 ± 11. Conversely, the NOE-restrained energy-minimized models exhibit unsatisfactory average correlation and RMSD scores of 9 ± 6 and 12 ± 6. A graphical presentation of the ANSURR results is reported in Supplementary Materials (Figure S3). The close Cα-RMSD values of the CS-Rosetta ensemble (1.53 ± 0.99 Å) and the NOE-restrained best cluster (1.57 ± 0.32 Å) seem to conflict with the RMSD scores of ANSURR that appear satisfactorily high, as expected for β-rich proteins, only with the CS-Rosetta ensemble. Also, the CSI-flexibility correlation score shows an appreciable difference between the CS-Rosetta and the NOE-restrained ensembles. Given the identity of the sequence and the associated chemical shift list, with the consequent flexibility estimates, the difference of CSI-flexibility correlation of the ANSURR assessments must be related to the different β-structure content of the two ensembles, namely the small deviations from regular geometry of the NOE-restrained ensemble shown in Figure 4 that prevent classification as β-structure and therefore conflict with local CSI. Even with a modest CSI-flexibility correlation score and a structural dispersion equivalent to that of the NOE-restrained best cluster, the CS-Rosetta cluster reaches the typically large RMSD score of the β-rich proteins.

No helical segments were identified from the ∆Δδ^13^Cα − ΔΔδ^13^Cβ chemical shift indexing analysis, although TALOS-N predicted four helical segments. Four of the NOE-restrained minimized structures have a right-handed helical fragment between residues 29 and 31. This fragment coincides with the putative CDR1 loop, and the recurrent three-residue helix in the structures could be an indication of a 3_10_-helical segment, which has a characteristic three-residue turn. The carbonyl oxygen of Thr28 (i) seems to face the HN of Ser31 (i + 3) at an average distance of 2.4 Å. The remaining structures have a helically-shaped loop at the same location; however, no secondary structure element came out for those structures. A similar helical segment is formed in eight of the ten structures of the NOE-restrained best cluster, between residues 62 and 64, with the carbonyl oxygen of Thr61 facing the HN of Val64. There is also a three-residue helix tract, i.e., a helical turn, where the carbonyl oxygen of Lys87 (i) seems to face the HN of Asp90 (i + 3) at an average distance of 2.1 Å, the residues completing a full turn. This is possibly also a 3_10_-helix. One segment in helical conformation is present in all of the NOE-restrained best cluster structures, in the supposed CDR3 loop, from position 107 to 111 (107–109 for one structure). This segment is in right-handed α-helix conformation, where the carbonyl oxygen of Thr107 (i) faces the HN of Thr111 (i + 4), at an average distance of 2.4 Å. The residues complete a full turn consistent with an α-helical segment. Another segment in helical conformation can be found in five of the structures between positions 113 and 115. This segment shows that the carbonyl oxygen of Arg112 (i) faces the HN of Asn115 (i + 3) at an average distance of 2.1 Å, i.e., a geometry that is consistent with a 3_10_-helix.

**Figure 6:**
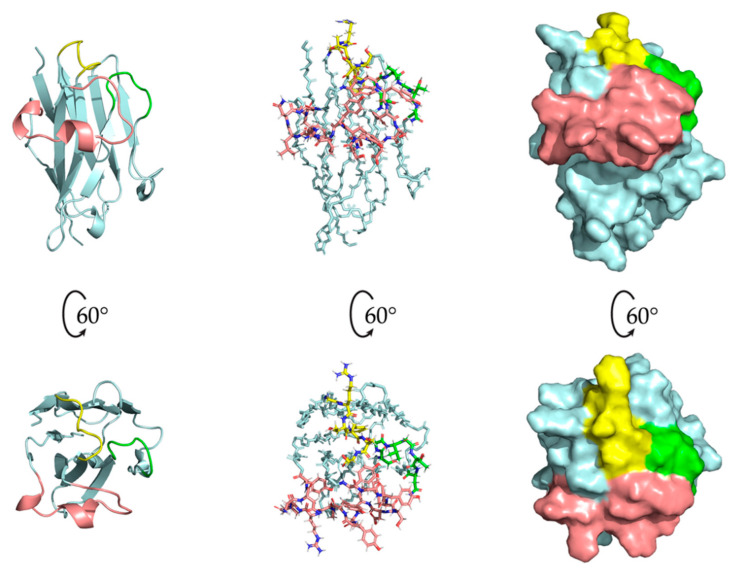
The CDRs of Nb23, with CDR1 in yellow, CDR2 in green, and CDR3 in salmon. The left column shows the cartoon representation of Nb23 without any sidechains. The central column shows the CDRs with sidechains (and only backbone for the β−core). The right column shows the surface of the protein with the CDRs highlighted. The predominance of the CDR3 in the antigen−binding site is evident, highlighting its importance in interacting with the antigen(s). Its orientation affects the size and shape of the antigen-binding site for the unbound nanobody, although the flexibility in residues 102−106 suggests that the CDR3 conformation may change as the nanobody binds its antigen(s).

**Figure 7:**
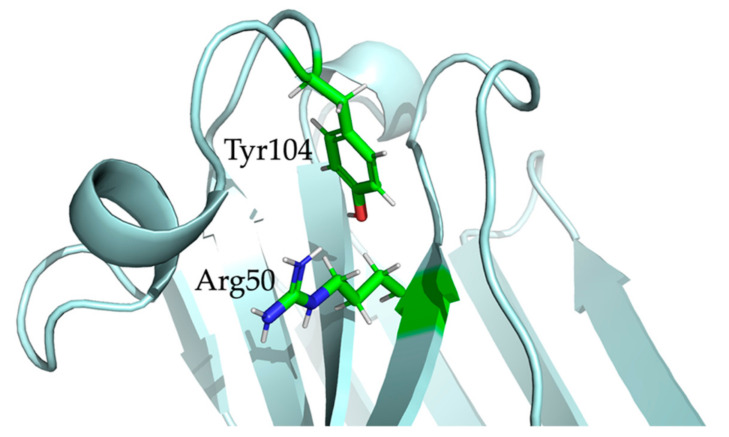
The Tyr104 phenolic ring in the CDR3 and the Arg50 βCH2 in β−strand C’’ of Nb23 show proximity as per the assigned NOE constraints. This indicates the possible presence of a guanidinium−π interaction, partially keeping the CDR3 in a defined orientation. The cartoon shows one of the arrangements of the residues in the NOE−restrained best cluster.

Figure 6 shows the orientation and surface of the CDR loops for the first structure of the NOE-restrained best cluster. The orientation of the CDR3 is of particular interest, given its length and the degree of mobility at the beginning of the loop evidenced by the ^15^N{^1^H} NOE analysis. Hence, several different orientations for the CDR3 were, in principle, possible. This is also reflected in the CS-Rosetta-generated models, where the β-core of the structure is very similar for each model while the CDR3 has a different conformation for each model. The CDR3 of the PONDEROSA-C/S energy-minimized structures included in the cluster has instead a more consistent conformation, with limited variations in the CDR3 relative to the CS-Rosetta models (Figure 5a,b). Fundamental to the orientation of the CDR3 in the NOE-restrained best cluster are the NOEs between Arg50 in β-strand C’ and Tyr104 of the CDR3. This well detectable interaction in the NOE spectra suggests a possible cation–π electrostatic interaction [30] between the Arg50 sidechain and the aromatic ring of Tyr104, which would partially keep the loop in a more defined orientation. Interestingly, position 104 of Nb24—the mentioned nanobody with similar binding properties to the β2m mutants as Nb23—is occupied by a cysteine which forms a disulfide bond with Cys33 of the β-strand C, essentially freezing the loop in a rigid conformation in Nb24. Position 33 is structurally arranged to be adjacent to position 50. Therefore, the cation–π interaction of Nb23 could vicariate the Cys33-Cys104 disulfide bridge of Nb24. One possible orientation of the sidechains of Arg50 and Tyr104 in Nb23 is shown in Figure 7, where the Arg50 sidechain faces the aromatic ring making the cation–π interaction possible [30].

### 2.6. Molecular Dynamics Simulations

**Figure 8:**
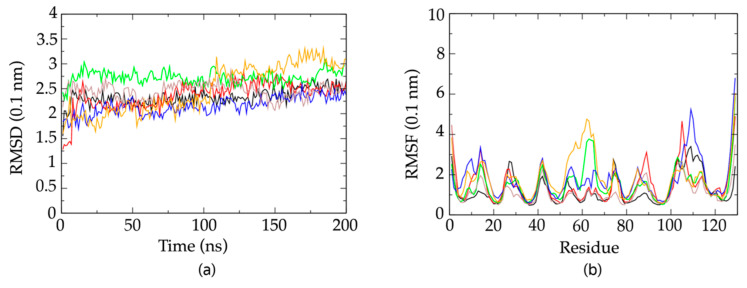
(**a**) RMSD with respect to the lowest energy structure of the NOE−restrained clusters as a function of time of the six MD simulations that were carried out starting from the minimized representative structures from the six clusters of the NOE−restrained PONDEROSA C/S models of Nb23. Black trace = cluster 1 (11 members); red trace = cluster 2 (2 members); green trace = cluster 3 (2 members); blue trace = cluster 4 (1 member); orange trace = cluster 5 (1 member); pale brown trace = cluster 6 (1 member). (**b**) RMSF in the same six MD simulations as in panel (**a**), as a function of the residue number of Nb23. The color code of the traces is the same as in panel (**a**).

The possible conformations for the CDR3 were investigated with molecular dynamics (MD) simulations, starting from representative of the six different clusters including all the best 18 energy-minimized structures from PONDEROSA C/S modeling. All simulations show an initial increase of the RMSD from the first structure of the specific NOE-restrained cluster, followed by rather stable equilibration at the value of about 2.5 Å (Figure 8a). During the simulation, most of the structures fluctuate about an average conformation with lower RMSD with respect to the initial structure, as witnessed by the much lower residue root mean square fluctuations (RMSFs) on the superimposed residues (Figure 8b). Large RMSF values are observed at loops and in the region 100–120 encompassing the CDR3. This is observed in most simulations, although in one of the simulations the region 50–70 is also showing large fluctuations.

MD confirms the proximity of Arg50 and Tyr104 sidechains in all of the simulations originating from the different clusters of PONDEROSA C/S energy-minimized conformers, with a geometry of either cation–π or π–stacking interaction in the snapshots of the simulation concerning the NOE-restrained best cluster.

An interesting observation is that the simulations starting from different minimized conformers of the PONDEROSA C/S clusters sample different regions of the conformational space, as can be seen by comparing the average RMSD at each residue for the ensemble of MD snapshots from each pair of simulations and for the ensemble of the pooled snapshots. An example is provided in Figure S4 with the pooling (dashed curve) of two of the MD snapshot ensembles depicted in Figure 8B. The large increase in RMSD upon pooling the two ensembles is indicative of large differences in the conformations about which the two MD simulations are fluctuating (see Figure S4).

## 3. Discussion

Nb23 was raised against ΔN6β2m to inhibit its amyloid formation, and could potentially be used for inhibiting fibril formation of other amyloidogenic β2m-variants. By using typical TROSY 3D experiments for backbone and aliphatic sidechain assignments, and 2D aromatic sidechain experiments for aromatic assignments, the chemical shifts of Nb23 were assigned. These chemical shift assignments were used for chemical shift-based homology modeling with CS-Rosetta giving a representative protein model as output. The model was in turn used together with the chemical shifts for NOE-restrained structure calculation supported by prior-knowledge of the structure. Relying on the experimental character of this prior knowledge, the choice was deliberately made to include only the unambiguously assigned NOEs to determine the solution structure of Nb23. Despite using what is considered a low number of NOE constraints (619) for structure determination—usually one would need ten NOEs per residue and Nb23 has ~130 residues—the resulting structures showed the general features of a single variable immunoglobulin domain and the general features of a nanobody. This minimalist approach was employed because of extensive signal overlap (especially for sidechains) making the unambiguous assignment not possible. Unfortunately, the issue of ambiguity could not be addressed because the necessary improvements of signal-to-noise and resolution conflicted with (i) the solubility and stability limits of Nb23 samples, which form precipitate in a matter of hours after dissolving the protein, and (ii) the current difficulties of accessing higher magnetic field facilities. Strictly speaking, the adopted minimalist approach is more rigorous than assigning NOEs, even when they are ambiguous, and then minimizing the constraints violations by progressive refinement with repeated trial-and-error calculations. When the spectral quality is not sufficient to remove assignment incompleteness or/and ambiguity, managing to reach the minimal restraint violation level with arbitrary release or retain of the internuclear distance attribution may only improve the precision of the determination, but definitely not its accuracy, as recently pointed out [29]. Thus, instead of relying on the number of NOE constraints as a quality determinant, the structures restrained with only unambiguous NOEs were evaluated on their similarities to the CS-Rosetta modelled ensemble, that was anyway based on the experimental chemical shifts (CS-Rosetta modelling included more than 700 chemical shift values constraining more than 350 dihedral angles).

The structures resulting from this protocol were subjected to energy minimization to adjust energetically unfavored sidechain conformations and to reduce the number of too-close contacts between adjacent atoms. A cluster of ten similar structures, deemed as representative of the structure of nanobody Nb23, was deposited in the PDB. The overall quality of this deposited ensemble was ranked to be far above average by the PDB validation server with respect to the deposited NMR structures (see Supplementary Materials).

The clustered structures were subjected to MD simulations to assess the conformational space available to the CDR3. The CDR3 showed particularly high values in RMSF, conforming that this functionally crucial region indeed could possibly have a range of conformations.

The deposited Nb23 structures (PDB ID 7EH3) have the main structural features observed in nanobodies: a β-core structure, and an extended CDR3, both for shielding solvent exposed hydrophobic sidechains (in particular Phe37, Phe47, Ile51, and Trp119) and for binding cryptic epitopes [1]. A comparative superposition of the solution structure of free Nb23 and the Nb24 structure to explain their activity differences can be misleading at the present stage. For Nb24, in fact, no structure of the free protein in solution is available as of now, whereas the crystal structures of the complexes with β2m variants were reported [11,12] to exhibit peculiar aspects that may be related to the crystalline state [11] or to the specifically selected β2m variant [12].

Structural characterization is fundamental to uncover subtle conformational differences that lead to changes in thermodynamic and kinetic parameters for the complexation of different nanobodies such as Nb23 and Nb24 with the β2m-mutants. In this respect, the lack of some fragments of secondary structure elements in the β-core of Nb23 is not of concern, because the departure from the canonical geometry amounts to small deviations that are consistent with loose arrangements and absence of inter-strand NOEs, especially at strand edges. This contributes to decreasing the number of employed NOE contacts, barely half of the required minimum threshold of ten contacts per residue. It was reasoned that the β-core of those immunoglobulin domains, so well represented in the PDB and in literature, would be well evidenced by the convergence of the CS-Rosetta models that guided the NOE search and could therefore determine a satisfactory result.

The impact of the ‘lacking’ β-strand content on the function of the nanobody should not be of great relevance, considering that the paratope of the nanobodies and immunoglobulin domains in general lies in the CDRs. Moreover, some loosening of the β-scaffold in the solution structure of isolated immunoglobulin domains is not surprising [8,10]. Of much more importance is instead the definition of the interactions that shape the CDR3 conformation, partially uncovered in this study. The structure and orientation of the CDR3 in Nb23 was found to both satisfy one of its principal tasks, i.e., shielding of conserved hydrophobic residues in the isolated protein, and be similar to that of the best CS-Rosetta model. In particular, Nb23 shows an interesting series of contacts between the sidechains of Arg50 and Tyr104 which could reflect the occurrence of a cation–π electrostatic interaction between the guanidinium and the phenolic ring. This interaction may vicariate for the disulfide bridge of Cys33 and Cys104 that occurs in camel-derived nanobodies such as Nb24. Besides the canonical disulfide linking the two β-sheets of immunoglobulins, camel-derived V_H_H domains exhibit in fact an additional cystine in the CDR3 region, that of course affects the local conformational options. Llama-derived V_H_H domains such as Nb23 do not possess this additional covalent constraint, but the occurrence of an energetically non-labile interaction such as a cation–π electrostatic one could help to modulate more precisely the available conformational repertoire. Importantly, the non-trivial character of this interaction should not conflict with the mobility in other regions of the CDR3, as suggested by the pattern of ^15^N{^1^H} NOE histogram (Figure 2) and the hypothesized conformational exchange that prevents the observation of the Ser105 NH signal.

In conclusion, Nb23′s structure determination is a first characterization step that will enable a more holistic assessment of its performance in inhibiting amyloidogenic β2m variants, once the solution structure of the isolated Nb24 and those of the complexes of both nanobodies with their antigens are also available. One possible outcome for this type of comparison could be the rational design of new hybrid nanobodies that perform better in fibril inhibition than the already existing ones.

## 4. Materials and Methods

### 4.1. Nb23 Expression and Labeling

Nb23 was previously obtained by immunization of a llama with a truncated version of β2-microglobulin, ΔN6β2-m (a β2-m variant devoid of the first six residues), as reported by Domanska et al. [11]. Nb23 was obtained uniformly doubly labeled with ^13^C and ^15^N by growing the transgenic *E. coli* strain containing the expression vector previously described [11] on ^13^C and ^15^N enriched medium. Expression and purification were performed by ASLA Biotech AB (Riga, Latvia), that also provided the unlabeled Nb23. Nb23 consists of 136 amino acids, including an initial Met introduced as a start codon for expression in *E. coli*, and a His6 tag at the C-terminus of the protein for purification purposes, amounting to a molecular weight of 15.1 kDa.

### 4.2. Nb23 Sample Preparation, NMR Data Acquisition, and Peak Assignment

All the NMR spectra were collected at the NMR facility of the Core Technology Platform at New York University Abu Dhabi on a 14 T Bruker Avance III spectrometer operating at 600, 150, and 60 MHz for ^1^H, ^13^C, and ^15^N, respectively, with a triple resonance cryoprobe. The acquisition temperature was always set to 298.2 K. All samples for backbone and sidechain assignment or homonuclear correlations were prepared at labeled or unlabeled protein concentrations ranging from 190 to 291 µM in 95/5 H_2_O/D_2_O and 10 mM phosphate buffer, pH 6.95, with or without NaCl (6.3–21 mM). Occasionally 19.5 mM bis-Tris aqueous buffer was also used, always at pH 6.95. The samples for aromatic sidechain assignment were prepared in D_2_O, at protein concentrations in the range 100–190 µM with 10 mM phosphate buffer, pH 6.98 (uncorrected pH-meter reading), without or with 20 mM NaCl. Importantly, the heteronuclear fingerprint of the ^15^N-^1^H HSQC spectra overlapped satisfactorily regardless of the mentioned buffer mixture. Protein concentrations were determined by UV absorption at 280 nm with an IMPLEN nanophotometer based on calculated molar extinction coefficients of 30,495 for Nb23. The sample concentrations were unstable over long time intervals. The initial concentration values invariably decreased by some 50% after 7–10 days as a consequence of protein precipitation. This proved detrimental for the sensitivity of the collected data sets, especially the later acquired ones, that could not be re-acquired due to labeled protein shortage.

A summary of the collected spectra with corresponding acquisition parameters is shown in Table 4. Pure phase detection in t1 and t2 dimensions of 3D data sets were obtained via gradient-based echo-antiecho selection and States-TPPI scheme [31,32,33]. The States-TPPI scheme was also employed for homonuclear NOESY and TOCSY spectra, whereas 2D heteronuclear spectra pure phase detection in t1 was obtained using echo-antiecho selection. The solvent was typically suppressed with a flip-back pulse [34], whereas in homonuclear spectra WATERGATE elements [35] applied in the excitation sculpting mode [36] were employed.

All 3D matrices were acquired with non-uniform sampling schemes by collecting 10%–20% of the whole datasets and by reconstructing the matrices with the dedicated routine of the Bruker Topspin 4.05 software [37]. The same software was used for processing all of the spectra with standard processing routines.

The NMR data were analyzed using NMRFAM-SPARKY [38], including peak assignment which was performed in a semi-automated manner using NMRFAM-SPARKY incorporated tools. The assignment list is available in BMRB, accession number 50808. Table 1 lists the overall assignment percentages.

### 4.3. Restrained Modeling

The set of the experimentally determined backbone and Cβ chemical shifts were input to run restrained MD modeling by means of the CS-ROSETTA server [19]. The chemical shifts represent experimental information that is employed to restrain the backbone dihedral angles *φ* and *ψ* by means of a pseudopotential term that introduces an energy penalty upon violation [19]. The same energy-penalty-driven approach was employed to calculate the structure based on the inter-proton distances obtained from the 2D and 3D NOESY spectra. The NOE-restrained structure determination was handled by means of the software suite PONDEROSA-C/S, using PONDEROSA-X refinement by which automated database-assisted NOE assignment is done (AUDANA algorithm) [50]. Experimentally determined chemical shift assignments for backbone, sidechain, and aromatic residues were input to automatically assign the 3D ^15^N-^1^H NOESY HSQC spectrum, and aliphatic and aromatic 3D ^13^C-^1^H NOESY HSQC spectra and calculate the structure as per the above procedure. Automated NOE-assignments were manually checked to remove ambiguous assignments and to add additional constraints. NOE intensities were considered only qualitatively as strong, medium and weak, corresponding to upper limit distances of 0.25, 0.35 and 0.5 nm, respectively.

### 4.4. Energy Minimization

The best 20 structures from the PONDEROSA C/S modeling were energy minimized first to remove the few (7.5 on average per each structure) bad contacts present, for 2000 minimization steps, using the steepest descent minimization algorithm. Since the solvent was not present at this stage, the GBSA implicit solvent model was adopted as implemented in the NAMD simulation software [51] according to the model by Onufriev, Bashford and Case [52]. Energy minimization resulted in structures devoid of bad contacts (according to the software Procheck [53]), except for two structures for which bad contacts persisted even after lengthening the minimization to 10,000 steps. The latter two structures were removed from the ensemble for MD simulations. At the same time, the ensemble of the ten most similar structures after energy minimization was retained as representing the NOE-restrained best cluster.

### 4.5. Molecular Dynamics Simulations

The best 18 structures resulting from energy minimization of the PONDEROSA C/S modeling were clustered by the PDB validation server (URL: www.wwpdb.org, accessed on 3 March 2021) into one 11-structure, two 2-structure and three 1-structure clusters. The best structure from each cluster was selected and subjected to MD simulations. Six MD simulations lasting 200 ns were performed using NAMD simulation software [51]. TIP3P water molecules (Jorgensen, 1983) and ions, to reach a 0.150 M ionic concentration, were added using the solvate module of the program VMD [54]. The simulation box was on average ca. 260,000 Å^3^ and the average number of atoms was 25,554. Molecular interactions were described by amber99sb-ildn force field [55]. Protein atoms were placed at the center of a cubic box at a minimum distance of 12 Å from the edge of the box. We used Periodic Boundary Conditions set by the size of the box. The solvated systems were energy minimized by 2000 steepest descent minimization steps. The equilibration phase was performed by increasing gradually the temperature from 0 to 310 K in 100 ps followed by further 900 ps. At this stage temperature was controlled by a simple velocity rescaling procedure and pressure at 1 atm was controlled by a pressure Langevin piston [56,57], with the period of 200.0 fs and decay constant of 100 fs. The time step was 1 fs, bonded interactions were computed every 1 fs and non-bonded interactions every 2 fs. Finally, MD simulation lasted 200 ns at constant pressure and temperature, the latter controlled through Langevin dynamics with damping constant of 1 ps^–1^. Snapshots were collected every 1 ns along the trajectory, giving a total of 200 snapshots which have been used in the analysis.

A total of 200 structures obtained from each MD simulation at 1ns time interval were analyzed as an ensemble of structures. The RMSD from the initial energy minimized structure was obtained by superimposing the backbone atoms of the residues structured in beta sheet based on multiple alignment of annotated sequences, i.e., residues 3–7, 10–12, 18–27, 34–39, 46–51, 55–60, 68–73, 78–83, 92–98. The time evolution of RMSD during the simulation was computed in the same way. From all pairwise snapshots superpositions, the root mean square fluctuations (RMSFs) for the backbone atoms of each residue were computed. The comparison between different simulations was performed by considering the ensemble of structures from each simulation and the ensemble obtained joining the two ensembles. A large increase in RMSF upon joining the two ensembles, compared to RMSFs observed in both ensembles, is indicative of local fluctuations about different conformations, i.e., the two simulations are sampling a different conformational space.

## Figures and Tables

**Table 1:** Chemical shift assignment completeness.

	Total	^1^H	^13^C	^15^N
**Backbone**	95%	96%	94%	96%
**Sidechain**	67%	73%	69%	0%
**Aromatic**	50%	68%	32%	0%
**Overall**	77%	80%	75%	71%

**Table 2:** Summary of features and violations for the CS-Rosetta ensemble, the NOE-restrained ensemble, and final NOE-restrained and energy minimized ensemble.

**Nb23 CS-Rosetta (10 Structures)**	
***Clashes***	
**van der Waals clashes**	8 (0.8 clashes/structure)
**Average clash**	0.48 ± 0.05 Å
***Ramachandran plot distribution***	
**Residues in favored regions**	97%
**Residues in allowed regions**	2%
**Outliers**	1%
**χ outliers per structure**	0.1
Cα-RMSD 1–129 w.r.t. lowest energy structure *	3.442 ± 2.212 Å
Cα-RMSD 1–102, 117–122 w.r.t. lowest energy structure *	1.531 ± 0.994 Å
**Nb23 NOE-Restrained (20 Structures)**	
***Distance Constraints***	
**Short-range**	417
**Medium-range**	16
**Long-range**	186
**Hydrogen bonds**	18
**Total**	637
***Violations***	
**Distance constraint violations**	115 (5.75 violations/structure)
**Short-range**	4
**Medium-range**	6
**Long-range**	76
**Hydrogen bonds**	29
**Average violation**	1.13 ± 0.61 Å
***Clashes***	
**van der Waals clashes**	189 (9.45 clashes/structure)
**Average clash**	0.48 ± 0.08 Å
***Ramachandran Plot Distribution***	
**Residues in favored regions**	93%
**Residues in allowed regions**	6%
**Outliers**	1%
**χ outliers per structure**	2.45
***Cα-RMSD 1–129 w.r.t. least violation structure***	1.98 ± 0.68 Å
***Cα-RMSD 3–100, 118–128 [Å] w.r.t least violation structure***	1.70 ± 0.68 Å
**Nb23 NOE-Restrained Energy-Minimized (10 Structures)**	
***Clashes***	
**van der Waals clashes**	0
***Ramachandran Plot Distribution***	
**Residues in favored regions**	96%
**Residues in allowed regions**	4%
**Outliers**	0%
**χ outliers per structure**	0.6
Cα-RMSD 1–129 w.r.t least violation structure *	1.57 ± 0.32 Å
Cα-RMSD 3–100, 118–128 w.r.t least violation structure *	1.23 ± 0.30 Å

* The pariwise Cα-RMSD for the respective ensembles, as well as the pairwise Cα-RMSD between the CS-Rosetta ensemble and the final NOE-restrained and energy minimized ensemble, are reported in Table S2 in the Supplementary Materials.

**Table 3:** β-structure content of the calculated Nb23 structures.

β-Strand	A	A *	B	C	C’	C’’	D	E	F	G	G *
Structure 1	3–7	-	17–25	32–39	46–51	-	69–73	77–84	92–100	-	123–125
Structure 2	3–7	-	17–25	32–39	46–51	-	69–73	77–84	92–100	117–119	-
Structure 3	3–7	-	17–25	32–39	46–51	-	69–73	77–84	92–100	117–119	123–125
Structure 4	3–7	-	17–25	32–39	46–51	-	69–73	77–84	93–100	-	-
Structure 5	3–7	-	17–25	32–38	46–51	-	69–73	77–84	93–99	-	-
Structure 6	3–7	-	17–25	32–39	46–52	-	69–73	77–84	93–100	117–119	-
Structure 7	3–7	-	17–25	32–39	46–51	-	69–73	77–84	93–101	117–119	-
Structure 8	3–7	-	17–26	32–39	46–51	59–61	69–73	77–84	92–100	117–119	123–125
Structure 9	3–7	-	17–25	32–39	46–51	-	69–73	77–84	93–100	117–119	-
Structure 10	3–7	-	17–25	32–39	46–51	-	69–73	77–84	93–100	117–119	-

* The A and G strands are composed of two separate β-segments as per the CSI and TALOS-N analyses. A dash (-) indicates the absence of a particular segment in the corresponding NOE-based Nb23 structures.

**Table 4:** List of the collected spectra for backbone and side-chain nuclei assignment of Nb23, with the corresponding acquisition parameters. Experiments denoted with tr indicate the use of TROSY pulse schemes.

Spectrum	Time Domain Dimensions	Transients (NS)	Carrier (ppm)	Spectral Width (ppm)	References
2D ^15^N^-1^H HSQC	t_2_ (^1^H): 2048 t_1_ (^15^N): 128	8, 16	t_2_ (^1^H): 4.7 t_1_ (^15^N): 118	t_2_ (^1^H): 16 t_1_ (^15^N): 50	[39]
2D tr^-15^N^-1^H HSQC	t_2_ (^1^H): 2048 t_1_ (^15^N): 80	16	t_2_ (^1^H): 4.7 t_1_ (^15^N): 118	t_2_ (^1^H): 16 t_1_ (^15^N): 50	[40]
3D tr-CBCANH	t_3_ (^1^H): 1024 t_2_ (^15^N): 50 t_1_ (^13^C): 128	576	t_3_ (^1^H): 4.7 t_2_ (^15^N): 118 t_1_ (^13^C): 43	t_3_ (^1^H): 14 t_2_ (^15^N): 50 t_1_ (^13^C): 80	[14,41]
3D tr-CBCA(CO)NH	t_3_ (^1^H): 1024 t_2_ (^15^N): 50 t_1_ (^13^C): 128	96	t_3_ (^1^H): 4.7 t_2_ (^15^N): 118 t_1_ (^13^C): 43	t_3_ (^1^H): 14 t_2_ (^15^N): 50 t_1_ (^13^C): 80	[14,42]
3D tr-HNCA	t_3_ (^1^H): 1024 t_2_ (^15^N): 50 t_1_ (^13^C): 96	32	t_3_ (^1^H): 4.7 t_2_ (^15^N): 118 t_1_ (^13^C): 54	t_3_ (^1^H): 18 t_2_ (^15^N): 50 t_1_ (^13^C): 80	[15]
3D tr-CC(CO)NH	t_3_ (^1^H): 1024 t_2_ (^15^N): 50 t_1_ (^13^C): 128	256	t_3_ (^1^H): 4.7 t_2_ (^15^N): 118 t_1_ (^13^C): 43	t_3_ (^1^H): 14 t_2_ (^15^N): 50 t_1_ (^13^C): 80	[14,43]
3D tr-H(CCO)NH	t_3_ (^1^H): 1024 t_2_ (^15^N): 50 t_1_ (^1^H): 128	256	t_3_ (^1^H): 4.7 t_2_ (^15^N): 118 ^1^H: 4.7	t_3_ (^1^H): 14 t_2_ (^15^N): 50 ^1^H: 14	[14,43]
3D tr-HBHA(CO)NH	t_3_ (^1^H): 1024 t_2_ (^15^N): 50 t_1_ (^1^H): 128	96	t_3_ (^1^H): 4.7 t_2_ (^15^N): 118 t_1_ (^1^H): 4.7	t_3_ (^1^H): 14 t_2_ (^15^N): 50 t_1_ (^1^H): 8	[14,44]
3D tr-HNCO	t_3_ (^1^H): 1024 t_2_ (^15^N): 50 t_1_ (^13^C): 96	32	t_3_ (^1^H): 4.7 t_2_ (^15^N): 118 t_1_ (^13^C): 173.5	t_3_ (^1^H): 14 t_2_ (^15^N): 50 t_1_ (^13^C): 22	[15]
3D tr-HN(CA)CO	t_3_ (^1^H): 1024 t_2_ (^15^N): 50 t_1_ (^13^C): 96	96	t_3_ (^1^H): 4.7 t_2_ (^15^N): 118 t_1_ (^13^C): 173.5	t_3_ (^1^H): 14 t_2_ (^15^N): 50 t_1_ (^13^C): 22	[45]
2D ^1^H^-1^H TOCSY	t_2_ (^1^H): 4096 t_1_ (^1^H): 768	192	t_2_ (_1_H): 4.7 t_1_ (_1_H): 4.7	t_2_ (_1_H): 14.4 t_1_ (_1_H): 14.4	[36,46,47]
3D ^15^N^-1^H NOESY HSQC	t_3_ (^1^H): 1024 t_2_ (^15^N): 50 t_1_ (^1^H): 400	96	t_3_ (^1^H): 4.7 t_2_ (^15^N): 122 t_1_ (^1^H): 4.7	t_3_ (^1^H): 14 t_2_ (^15^N): 42 t_1_ (^1^H): 14	[31,39,48]
2D CBHD (D2O)	t_2_ (^1^H): 2048 t_1_ (^13^C): 98	1024	t_2_ (^1^H): 4.7 t_1_ (^13^C): 36	t_2_ (^1^H): 16 t_1_ (^13^C): 28	[16]
2D CBHE (D2O)	t_2_ (^1^H): 2048 t_1_ (^13^C): 98	1024	t_2_ (^1^H): 4.7 t_1_ (^13^C): 36	t_2_ (^1^H): 16 t_1_ (^13^C): 28	[16]
2D ^1^H^-1^H NOESY (D2O)	t_2_ (^1^H): 4096 t_1_ (^1^H): 400	192	t_2_ (^1^H): 4.7 t_1_ (^1^H): 4.7	t_2_ (^1^H): 14.4 t_1_ (^1^H): 14.4	[36,46,49]
3D ^13^C^-1^H NOESY HSQC aliphatic (D2O)	t_3_ (^1^H): 1024 t_2_ (^13^C): 80 t_1_ (^1^H): 160	96	t_3_ (^1^H): 4.7 t_2_ (^13^C): 43 t_1_ (^1^H): 4.7	t_3_ (^1^H): 14 t_2_ (^13^C): 80 t_1_ (^1^H): 14	[31,39,48]
3D ^13^C^-1^H NOESY HSQC aromatic (D2O)	t_3_ (^1^H): 1024 t_2_ (^13^C): 80 t_1_ (^1^H): 160	96	t_3_ (^1^H): 4.7 t_2_ (^13^C): 105 t_1_ (^1^H): 4.7	t_3_ (^1^H): 14 t_2_ (^13^C): 80 t_1_ (^1^H): 14	[31,39,48]
2D ^13^C^-1^H HSQC	t_2_ (^1^H): 1024 t_1_ (^13^C): 196	32	t_2_ (^1^H): 4.7 t_1_ (^13^C): 72	t_2_ (^1^H): 16 t1 (^13^C): 165	[39]

## Data Availability

The data presented in this study are openly available in the Biological Magnetic Resonance Bank (BMRB), accession number 50808, and in the Protein Data Bank (PDB), PDB ID 7EH3.

## Supplementary Materials

The following supplementary material is available online. Supplementary information (Validation, Energy minimization, Assignment and Structure data); Table S1: H-bond list; Table S2: Pairwise RMSD; Figure S1: CD spectroscopy of Nb23 in H_2_O; Figure S2: CD spectroscopy of Nb23 in H_2_O with TCEP; Figure S3: ANSURR assessment for the CS-Rosetta and the NOE-restrained best cluster; Figure S4: RMSF at each residue of Nb23 in the MD simulation.

## Sample Availability

Samples of the compounds are available from the authors.
