# Supplementary material for: Structure of Nanobody Nb23
Source: Molecules. 2021 Jun 11;26(12):3567. doi: 10.3390/molecules26123567 (PMC8230604; doi:10.3390/molecules26123567)
Supplement: Supplementary file 1 [file molecules-26-03567-s001.zip › molecules-1201620-supplementary.pdf]

Structure of nanobody Nb23

Mathias Percipalle [1,2], Yamanappa Hunashal [1], Jan Steyaert [3,4], Federico Fogolari [5,6], and Gennaro Esposito [1,6*]

[1] Science Division, New York University, Abu Dhabi, UAE; mp5604@nyu.edu yh45@nyu.edu ge22@nyu.edu
[2] Department of Chemistry and Magnetic Resonance Center (CERM), University of Florence, Florence, Italy
[3.] Structural Biology Brussels, Vrije Universiteit Brussel (VUB), Brussels, Belgium; jan.steyaert@vub.be
[4.] VIB-VUB Center for Structural Biology, VIB, Brussels, Belgium
[5.] DMIF, Udine University, Udine, Italy; federico.fogolari@uniud.it
[6.] INBB, Roma, Udine, Italy
* Correspondence: ge22@nyu.edu

SUPPLEMENTARY MATERIALS

1. Supplementary information

1.1 Nb23 Structure Validation

The resulting 20 best structures from the NOE-restrained modeling carried out with PONDEROSA-C/S were validated through the wwwPDB Validation Service [26], which uses MolProbity [27, 28] to check for major geometrical issues in the models the wwPDB validation server identified 189 too-close contacts (overlapping van der Waals radii) between atoms for the ensemble of 20 models, with 123 unique clashes. This translates to about nine to ten clashes per model. Overlaps of van der Waals radii of non-polar atoms are considered unfavorable when exceeding 0.4 Å [28]. The most prominent and recurring clashes were located in the mobile region of the CDR3 (Tyr101). For the backbone conformation of the ensemble of structures, 93 %of the residues were located in favored Ramachandran regions and 6 % in allowed regions. Out of 32 (1 %) Ramachandran plot outliers, 18 were unique, the most frequent being Tyr103 present in five of the models. Sidechain conformation outliers were also present, with a total of 54 residues having a deviating χ torsion angle, the most frequent one being Leu11 present in 17 of the 20 models. Nevertheless, in terms of structure quality, the determined Nb23 ensemble of 20 structures performed better than average in all above respects compared with all NMR structures in the PDB.

1.2 Nb23 NOE-restrained best cluster

In the ten-structure cluster that were retained, minimization introduced five unique bond angle outliers, the worst deviating by 3.34° from the ideal angle, which is not a very important deviation. However, after minimization, the total number of too-close contacts was zero. Ramachandran favored residues reached 96 % and the number of outliers was six (five unique), the most prominent remaining Tyr103 (two models). The number deviating χ torsion angle was 26 after minimization, with 12 unique outliers, and the most reoccurring outlier (Leu11) had a frequency of nine. The validation report of the Nb23 NOE-restrained best cluster is available in the PDB (https://www.rcsb.org/), PDB ID 7EH3.

1.3 Nb23 assignment and structures

The assignment list for Nb23 is available in BMRB (https://bmrb.io/), accession number 50808. The structure of Nb23 is available in the PDB (https://www.rcsb.org/), PDB ID 7EH3. The validation report of the Nb23 NOE-restrained best cluster is available at the same address.

2. Tables

Table S1. Hydrogen bonds used for Nb23 structure calculation

Residue	Location in structure
Met34	C-strand
Ala35	C-strand
Trp36	C-strand
Phe37	C-strand
Arg38	C-strand
Gln39	C-strand
Lys43	Loop connecting C- and C′-strand
Arg50	C′-strand
Ile69	D-strand
Ser71	D-strand
Asp73	D-strand
Val79	E-strand
Leu81	E-strand
Met83	E-strand
Ser85	Loop connecting E- and F-strand
Tyr118	G-strand
Thr123	G-strand
Val125	G-strand

Table S2. Pairwise intra-set and inter-set Cα-RMSD of and between the CS-Rosetta and final NOE-restrained and energy minimized ensembles for the residues deemed as non-flexible and for the β-strands of the first 100 residues in CS-Rosetta (deemed as the most well defined in that ensemble).

**Nb23 CS-Rosetta (10 structures)**	**Cα-RMSD**
1-129	3.72 ± 0.98 Å
1-102, 117-122	1.71 ± 0.20 Å
3-7, 10-12, 18-27, 34-39, 46-51, 55-60, 68-73, 78-83, 92-98	1.24 ± 0.26 Å
**Nb23 NOE-restrained Energy-minimized (10 structures)**	
1-129	1.65 ± 0.33 Å
3-100, 118-128	1.33 ± 0.34 Å
3-7, 10-12, 18-27, 34-39, 46-51, 55-60, 68-73, 78-83, 92-98	1.17 ± 0.34 Å
**CS-Rosetta ensemble and NOE-restrained Energy-minimized ensemble**	
1-129	4.12 ± 0.69 Å
1-102, 117-122	3.26 ± 0.69 Å
3-100, 118-128	3.06 ± 0.30 Å
3-7, 10-12, 18-27, 34-39, 46-51, 55-60, 68-73, 78-83, 92-98	2.59 ± 0.25 Å

3. Figures

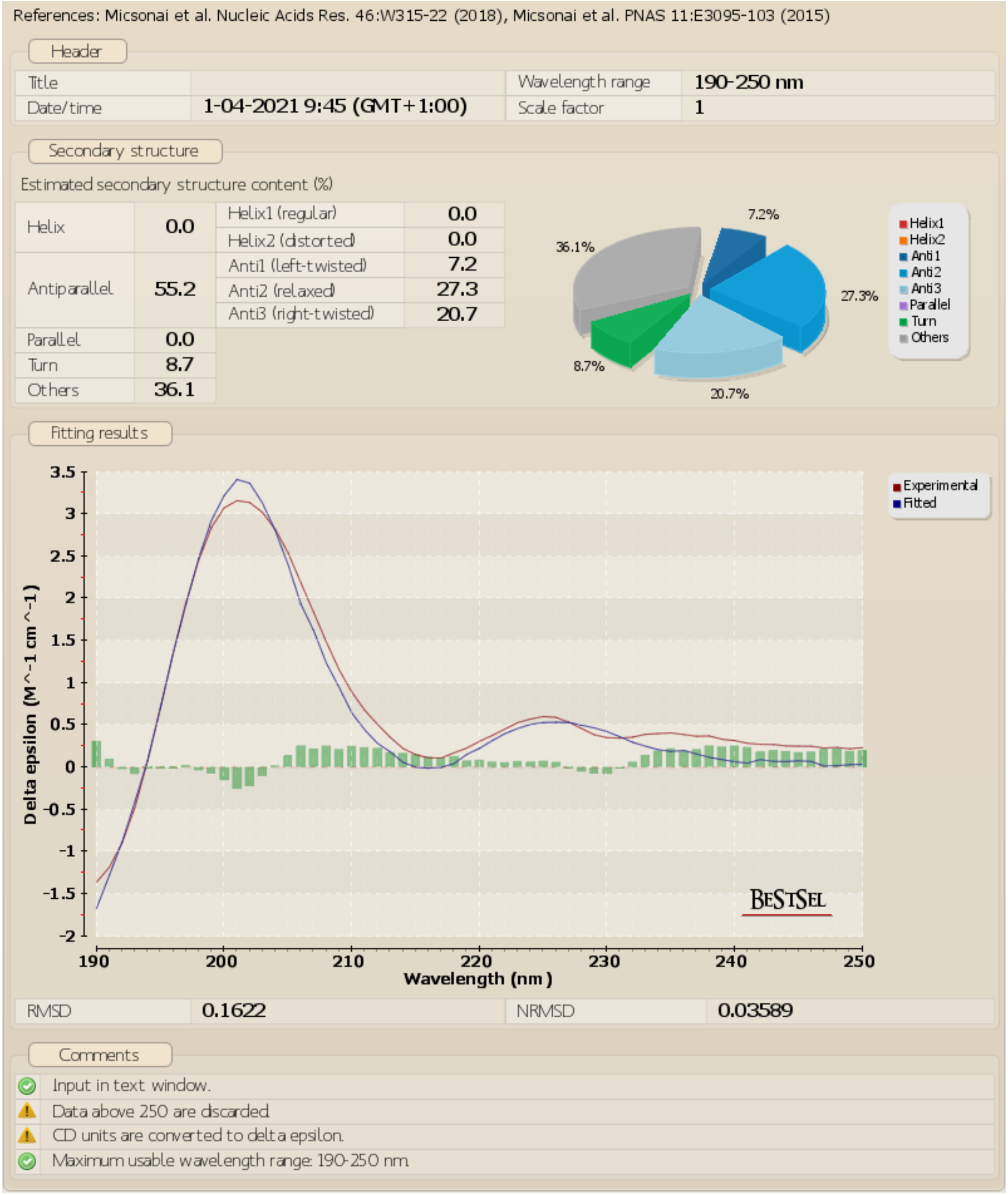

Figure S1. CD spectroscopy results for Nb23 5µM in H_2O from BeStSel, a server for CD spectroscopy secondary structure analysis particularly suited for proteins rich in β-structures. The structure is predominantly β, with 55.2% in antiparallel β-strand conformation, and more than half of the β-content in twisted β-strand conformation. No helices were identified. The closest matching CATH categories obtained by BeStSel when searching the PDB in a subset filtered for ≤ 90 % sequence homology where Mainly Beta (Class), Sandwich (Architecture), and Immunoglobulin-like (Topology), which are typical categories of nanobody structures.

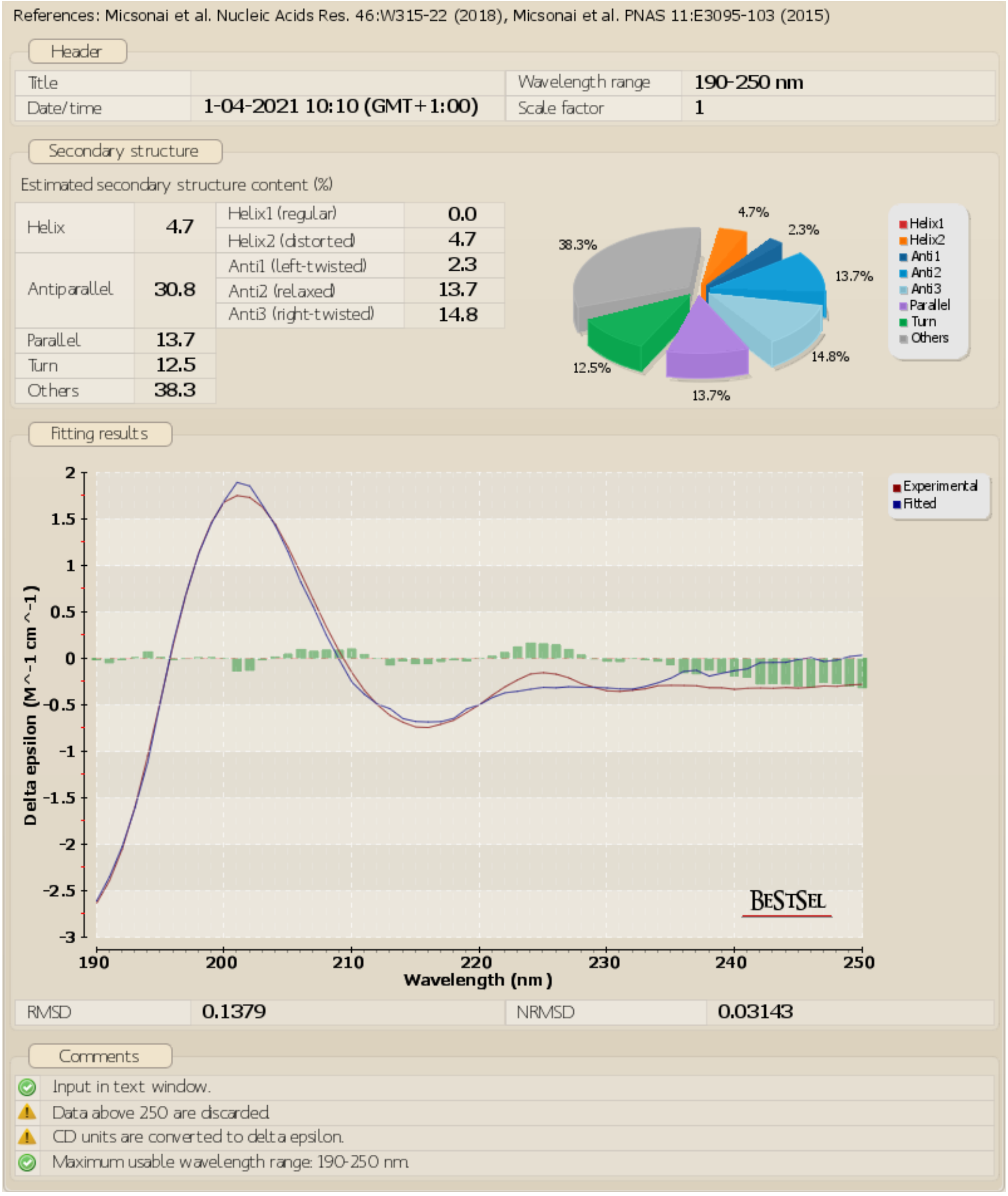

Figure S2. CD spectroscopy results for Nb23 5μM with the reducing agent TCEP 20μM and in H_2O from BeStSel, a server for CD spectroscopy secondary structure analysis particularly suited for proteins rich in β-structures. The β-content is diminished to 44.5% β-strand, suggesting the breach of the disulfide bridge between Cys22 and Cys96.

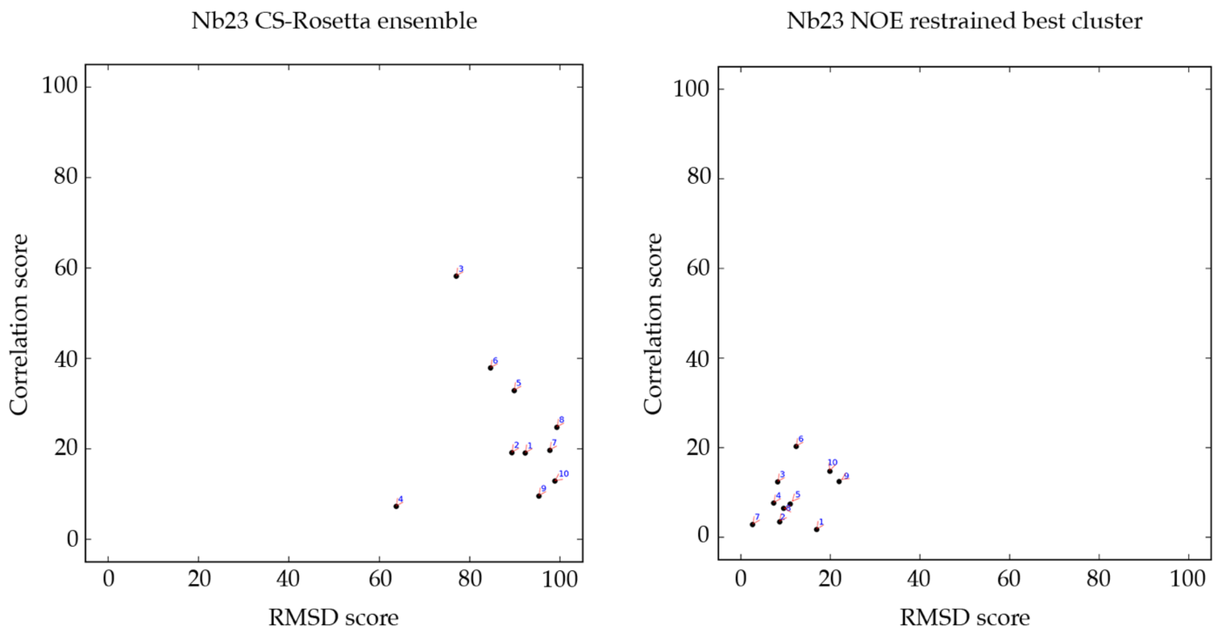

Figure S3. Structure accuracy assessment according to ANSURR criteria for the ensembles of Nb23 conformers from CS-Rosetta modeling and NOE-restrained best cluster from PONDEROSA C/S modeling.

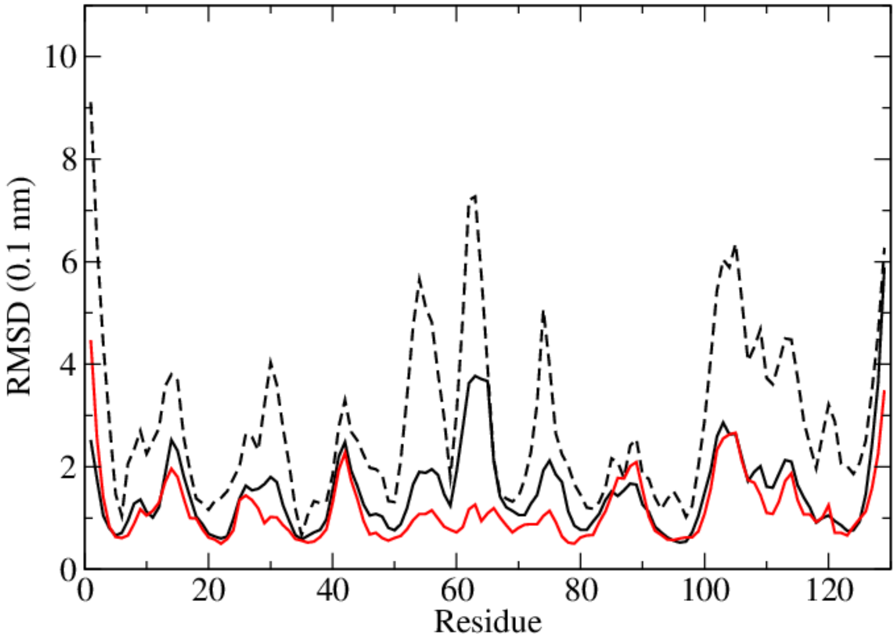

Figure S4. RMSF at each residue of Nb23 in the MD simulation starting from lowest energy structure of the NOE-restrained best clusters 3 (black trace) and 6 (red trace). The dashed trace is obtained from computing the RMSF on the pooled trajectories. Similar results can be obtained by comparing any pair of RMSF data in panel B with the RMSF computed on the pooled respective trajectories.
